# Longitudinal Analysis of Mpox Virus DNA Detectability From Multiple Specimen Types During Acute Illness: A Cohort Study

**DOI:** 10.1093/ofid/ofae073

**Published:** 2024-02-07

**Authors:** Darrell H S Tan, Oscar Pico Espinosa, John Matelski, Shreya S Khera, Attia Qamar, Reva Persaud, Jacklyn R Hurst, Angel Ly, Jessica Lam, Maedeh Naghibosadat, Natasha Christie, Maan Hasso, Kevin Gough, Linda R Taggart, Charlie Tan, Mario Ostrowski, Huiting Ma, Scott D Gray-Owen, Robert Kozak, Sharmistha Mishra

**Affiliations:** Division of Infectious Diseases, St. Michael's Hospital, Toronto, Ontario, Canada; MAP Centre for Urban Health Solutions, St. Michael's Hospital, Toronto, Ontario, Canada; Department of Medicine, University of Toronto, Toronto, Ontario, Canada; Institute of Medical Science, University of Toronto, Toronto, Ontario, Canada; Institute of Health Policy, Management and Evaluation, Dalla Lana School of Public Health, Toronto, Ontario, Canada; MAP Centre for Urban Health Solutions, St. Michael's Hospital, Toronto, Ontario, Canada; Division of Infectious Diseases, St. Michael's Hospital, Toronto, Ontario, Canada; MAP Centre for Urban Health Solutions, St. Michael's Hospital, Toronto, Ontario, Canada; Scarborough Health Network, Scarborough, Ontario, Canada; MAP Centre for Urban Health Solutions, St. Michael's Hospital, Toronto, Ontario, Canada; Biological Sciences, Sunnybrook Research Institute, Sunnybrook Hospital, Toronto, Ontario, Canada; Toronto High Containment Facility, Temerty Medicine, University of Toronto, Toronto, Ontario, Canada; Toronto High Containment Facility, Temerty Medicine, University of Toronto, Toronto, Ontario, Canada; Biological Sciences, Sunnybrook Research Institute, Sunnybrook Hospital, Toronto, Ontario, Canada; Toronto High Containment Facility, Temerty Medicine, University of Toronto, Toronto, Ontario, Canada; Public Health Ontario Laboratory, Toronto, Ontario, Canada; Division of Infectious Diseases, St. Michael's Hospital, Toronto, Ontario, Canada; Department of Medicine, University of Toronto, Toronto, Ontario, Canada; Division of Infectious Diseases, St. Michael's Hospital, Toronto, Ontario, Canada; Department of Medicine, University of Toronto, Toronto, Ontario, Canada; Division of Infectious Diseases, St. Michael's Hospital, Toronto, Ontario, Canada; Department of Medicine, University of Toronto, Toronto, Ontario, Canada; Division of Infectious Diseases, St. Michael's Hospital, Toronto, Ontario, Canada; Department of Medicine, University of Toronto, Toronto, Ontario, Canada; MAP Centre for Urban Health Solutions, St. Michael's Hospital, Toronto, Ontario, Canada; Toronto High Containment Facility, Temerty Medicine, University of Toronto, Toronto, Ontario, Canada; Department of Molecular Genetics, Temerty Medicine, University of Toronto, Toronto, Ontario, Canada; Biological Sciences, Sunnybrook Research Institute, Sunnybrook Hospital, Toronto, Ontario, Canada; Department of Laboratory Medicine and Pathobiology, University of Toronto, Toronto, Ontario, Canada; Division of Infectious Diseases, St. Michael's Hospital, Toronto, Ontario, Canada; MAP Centre for Urban Health Solutions, St. Michael's Hospital, Toronto, Ontario, Canada; Department of Medicine, University of Toronto, Toronto, Ontario, Canada; Institute of Medical Science, University of Toronto, Toronto, Ontario, Canada; Institute of Health Policy, Management and Evaluation, Dalla Lana School of Public Health, Toronto, Ontario, Canada; ICES, Toronto, Ontario, Canada

**Keywords:** monkeypox, mpox, orthopoxvirus, polymerase chain reaction, prospective cohort study, tecovirimat

## Abstract

**Background:**

Longitudinal data on the detectability of monkeypox virus (MPXV) genetic material in different specimen types are scarce.

**Methods:**

We describe MPXV-specific polymerase chain reaction (PCR) results from adults with confirmed mpox infection from Toronto, Canada, including a cohort undergoing weekly collection of specimens from multiple anatomic sites until 1 week after skin lesions had fully healed. We quantified the time from symptom onset to resolution of detectable viral DNA (computed tomography [Ct] ≥ 35) by modeling exponential decay in Ct value as a function of illness day for each site, censoring at the time of tecovirimat initiation.

**Results:**

Among 64 men who have sex with men, the median (interquartile range [IQR]) age was 39 (32.75–45.25) years, and 49% had HIV. Twenty received tecovirimat. Viral DNA was detectable (Ct < 35) at baseline in 74% of genital/buttock/perianal skin swabs, 56% of other skin swabs, 44% of rectal swabs, 37% of throat swabs, 27% of urine, 26% of nasopharyngeal swabs, and 8% of semen samples. The median time to resolution of detectable DNA (IQR) was longest for genital/buttock/perianal skin and other skin swabs at 30.0 (23.0–47.9) and 22.4 (16.6–29.4) days, respectively, and shortest for nasopharyngeal swabs and semen at 0 (0–12.1) and 0 (0–0) days, respectively. We did not observe an effect of tecovirimat on the rate of decay in viral DNA detectability in any specimen type (all *P* > .05).

**Conclusions:**

MPXV DNA detectability varies by specimen type and persists for over 3–4 weeks in skin specimens. The rate of decay did not differ by tecovirimat use in this nonrandomized study.

Mpox is a systemic infection caused by monkeypox virus (MPXV), a DNA orthopoxvirus that has been re-emerging in West and Central Africa for years [1–3]. Infection typically involves flu-like symptoms, tender lymphadenopathy, and polymorphic skin lesions [4, 5]. A large international mpox outbreak began in May 2022, resulting in 171 deaths and >92 000 cases in 116 countries as of December 22, 2023 [6]. During the global epidemic, immunization with modified vaccinia Ankara-Bavarian Nordic (MVA-BN; or Imvamune) vaccine was found to reduce the risk of infection, with effectiveness depending on the number of doses and time since vaccination [7, 8]. Tecovirimat, an oral antiviral with anti-orthopoxvirus activity [9], was used to treat severe cases as well as those at risk of severe disease [4].

The 2022 global epidemic was concentrated in sexual networks of gay, bisexual, and other men who have sex with men (GBM) [10, 11]. Transmission largely occurred through direct contact with infected skin lesions and mucosal sites during sexual encounters, such that proctitis, pharyngitis, and anogenital lesions were common [4]. Public health guidance generally recommends that persons with infection self-isolate or, at minimum, keep skin covered until lesions have completely healed over and revealed fresh, epithelialized skin, a process that often takes weeks to occur. Due to concerns about onward sexual transmission, the World Health Organization recommended that condoms be used for an even longer period of 12 weeks after recovery from illness [12].

To date, data are limited regarding the extent to which virus or viral genetic material can be detected in various anatomic compartments and corresponding biospecimens during this period. While numerous case series have shown that viral DNA can sometimes be detected in various clinical specimens even weeks after symptom onset [13–15], very few studies have prospectively evaluated viral DNA detectability in a systematic, longitudinal fashion [16, 17]. Characterizing the kinetics of MPXV detectability during infection is important for understanding the diagnostic yield of different specimen types at various stages of infection and could aid patients and clinicians in interpreting test results. We present a descriptive analysis of quantitative MPXV DNA detection over time across multiple specimen types in adults with mpox infection. Our primary objective was to quantify the time from symptom onset to resolution of detectable viral DNA during acute mpox infection by specimen type. Our secondary objective was to estimate the proportion of participants with detectable DNA at study entry and at 1 week after complete clinical resolution by specimen type. In exploratory analyses, we investigated the relationships between the presence of pharyngitis and proctitis symptoms, as well as receipt of tecovirimat, and DNA detectability.

## METHODS

### Design and Participants

We analyzed data from 2 samples of adult study participants recruited between June and October 2022. The first comprises participants in the Mpox Prospective Observational Cohort Study (MPOCS), an ongoing cohort study of individuals with confirmed mpox infection that involves systematic, longitudinal collection of biological samples (herein, “cohort” participants). We also include data on a second group (herein, “case series” participants) diagnosed with mpox at the same study center who declined participation in the cohort but consented to the abstraction of data from medical records. Eligibility criteria for both studies involved only a clinical suspicion of mpox infection at the time of enrollment with no specific exclusion criteria; for this report, we report on only those with microbiologically confirmed infection. The initial target sample size was 100 participants for each of the 2 contributing studies, based on financial and feasibility considerations, although the number ultimately enrolled was limited due to a decline in the local epidemic.

During the study period, tecovirimat was available in limited supply in Ontario. Clinicians could request compassionate release of drug from a provincial stockpile, although use was restricted to those with severe manifestations owing to limited supply. The recommended dosage was 600 mg twice daily for 14 days [18].

### Study Procedures—Cohort Participants

MPOCS participants underwent a baseline clinical assessment by an infectious diseases physician researcher and a trained research coordinator as soon as possible after the diagnosis was suspected by a health care provider. This visit included an interview and participant questionnaire for characterization of the participant's illness, epidemiologic exposure history, medical history, and demographics. Personal protective equipment including an N95 mask, face shield, gown, and gloves were used for all encounters.

Study staff collected biological samples for MPXV detection by quantitative polymerase chain reaction (qPCR) from 6 different anatomic compartments, regardless of the presence/absence of symptoms at that site, including nasopharyngeal swabs, pharyngeal swabs, rectal swabs, skin swabs, urine, and semen. When reporting skin swab results, we distinguish between swabs of genital/buttock/perianal skin and swabs of all other skin sites. All swabs were collected by trained study staff into vials containing viral transport medium. Urine and semen samples were self-collected by participants into separate sterile containers. All specimens were refrigerated at 4°C while awaiting transportation to the study storage facility and frozen at −80°C within 24 hours of collection.

In-person follow-up visits for MPOCS were conducted by study staff weekly (±3 days) until 1 week after the participant's final cutaneous lesions had fully resolved, defined as the time when scabs had fallen off to reveal fully epithelialized skin, as determined by the patient's physician. Activities at each follow-up visit included an updated medical history and repeat biological sampling from the same compartments. Participants received $100 Canadian dollars at baseline and $75 Canadian dollars per follow-up visit.

For samples collected on cohort participants, the presence of MPXV DNA was determined using a real-time PCR pan-orthopoxvirus assay that amplified the E9L gene. Nucleic acids were extracted from all collected samples using the QIAamp Viral RNA Mini Kit (Qiagen, Hilden, Germany) according to the manufacturer's protocol for purification of viral nucleic acids from plasma, serum, and cell-free body fluids [19]. After the wash steps, columns were eluted twice with a 40-μL buffer AVE to increase viral nucleic acid yields and stored at −80°C until use.

Eluates were run on the Rotorgene Q platform (Qiagen, Hilden, Germany) with cycling conditions of 60°C for 1 minute, 95°C for 2 minutes, followed by 40 amplification cycles of 95°C for 10 seconds and 56°C for 40 seconds. A total 20-μL reaction mixture contained Luna Probe One step reaction mix (NO ROX) and Luna WarmStart RT Enzyme Mix (New England Biolabs Inc.), 10 μM of E9L forward (5′-TCA ACT GAA AAG GCC ATC TAT GA-3′), 10 μM of E9L reverse (5′- GAG TAT AGA GCA CTA TTT CTA AAT CCC A-3′), 10 μM of E9L MPXV probe (5′-6FAM-CCA TGC AAT ATA CGT ACA AGA TAG TAG CCA A-BHQ1-3′), 10 μM of RNAse polymerase forward (5′-AGA TTT GGA CCT GCG AGC G-3′), 10 μM of RNAse polymerase reverse (5′-GAG CGG CTG TCT CCA CAA GT-3′), 10 μM of RNAse polymerase probe (5′-Quasar-705-TTC TGA CCT GAA GGC TCT GCG CG-BHQ2-3′), and 5 μL of template DNA from nucleic acid extractions. In each PCR run, a negative control and positive control containing MPXV DNA extracted from a known MPXV-positive clinical sample were included, as well as known serial dilutions of pUC57 plasmid containing a 365-bp insertion of the E9L gene sequence. Quantification of MPXV DNA (copies) in each specimen was derived from standard curves of known E9L gene copy numbers from pUC57-E9L dilutions and their computed tomography (Ct) values in each run.

### Study Procedures—Case Series Participants

Case series participants underwent clinical assessment and biological sampling as determined by their treating physician. Data were extracted from medical records retrospectively by a trained chart abstractor. Specimen collection and clinical follow-up were under the direction of the treating physician, with samples transported to and processed at the Public Health Ontario Laboratory within 24 hours of collection.

For case series participants, the presence of MPXV DNA was determined using a real-time PCR assay that amplified the MPXV G2R gene, an MPXV clade II–specific target, and an RNaseP extraction control, using established protocols described previously [20].

### Data Analysis

Demographic and clinical characteristics of study participants were summarized descriptively. We compared characteristics of cohort and case series participants using the Wilcoxon rank-sum test or Fisher exact test as appropriate.

As noted above, qPCR was performed by 2 different laboratories for the cohort and case series participants, with the more numerous cohort assays amplifying the E9L gene and the case series assays yielding either pan-mpox or clade 2 values. We identified 11 specimens that were tested on all platforms and found Pearson's correlation coefficients with E9L of 0.54 (MPXV G2R or pan-mpox assay) and 0.62 (clade II–specific assay). To standardize assay values, we used linear regression to impute E9L values for the case series participants using either pan-mpox or clade 2 assay results, as available, in order to facilitate the inclusion of all the available laboratory data in our statistical models.

For our primary analyses, we modeled exponential decay in the E9L Ct value as a function of day since symptom onset separately for each specimen type using linear mixed effects regression (LMER). For patients who received tecovirimat, only values before treatment initiation were included. The natural log of the E9L Ct value was regressed on days since symptom onset, and a random intercept was included to account for patient-level clustering. We report a visualization of each model along with the *P* value testing whether the rate of change in the expected mean E9L Ct value was significantly different from 0. We used these models to assess “days since symptom onset when the expected E9L Ct value equals 35,” as well as the range of days when the 95% CI for the expected E9L covers 35. We selected a Ct threshold of ≥35 for defining clearance of viral DNA based on prior reports suggesting that this or lower Ct values correspond to a lower likelihood of viral infectivity [21–24]. To address potential bias owing to imbalanced data (participants with only a single testing date were typically seen early during follow-up), we further conducted sensitivity analyses in which participants who contributed data for a given specimen type on only a single date were excluded.

In secondary analyses, we quantified the proportion of participants with detectable viral DNA at the first sampling time point. We further quantified the proportion of cohort study participants still yielding detectable viral DNA at the final sampling time point (1 week after healing of the last skin lesion). These analyses were stratified by specimen type and excluded any samples collected after tecovirimat initiation.

In exploratory analyses, we examined the relationship between clinical symptoms and specimen type–specific detectability of viral DNA by comparing the results of nasopharyngeal or pharyngeal swabs between participants who ever experienced symptoms of pharyngitis during their illness. Similarly, we compared rectal swab results among those who ever experienced proctitis.

Finally, we assessed the impact of tecovirimat on viral DNA detectability using mixed log-linear models in which the outcome was equal to the Ct value and the primary predictor variable was an interaction between days since symptom onset and treatment with tecovirimat as a time-varying covariate. We used these models to assess group differences in the mean time to resolution for patients who never received tecovirimat treatment compared with those with any tecovirimat during follow-up.

All statistical testing and modeling were performed using R, version 4.2.1 [25], with alpha = .05.

## RESULTS

Between June 20, 2022, and August 25, 2022, we enrolled 26 MPOCS and 39 case series participants; 1 MPOCS participant subsequently withdrew, leaving 64 participants overall (Supplementary Figure 1). There were no significant differences in the demographic or clinical characteristics of the 2 groups at baseline, but the interval between symptom onset and study baseline was longer for cohort than case series participants (median, 15 vs 6 days; *P* < .001), perhaps due to the extra time required for referral and cohort enrollment activities or due to other unidentified factors (Table 1). All self-identified as GBM, 49% had HIV, and 33% were using HIV pre-exposure prophylaxis. The median (interquartile range [IQR]) age was 39 (32.75–45.25) years. Among HIV-positive participants, 90% had plasma HIV RNA <20 copies/mL, and the median (IQR) CD4 count was 467.5 (335.75–677.75) cells/mm^3^. Zero participants were receiving tecovirimat at baseline, and 12/56 (21%) with available data had received at least 1 dose of MVA-BN vaccine before enrollment.

**Table 1.** Demographic and Clinical Characteristics of Study Participants^a^

Characteristic	Overall (n = 64)	MPOCS (n = 25)	Case Series (n = 39)	*P* Value^b^
Age, y	39.0 (32.75–45.25)	37 (33–42)	40 (32.5–46)	.84
Male sex assigned at birth	64 (100)	25 (100)	39 (100)	N/A
Male gender identity	64 (100)	25 (100)	39 (100)	N/A
Ethnoracial identity				
White	22 (34)	9 (36)	13 (33)	.40
Black	10 (16)	5 (20)	5 (13)	
East/Southeast Asian	7 (11)	5 (20)	2 (5)	
South Asian	4 (6)	1 (4)	3 (8)	
Latinx	16 (25)	4 (16)	12 (31)	
Another identity not listed here	3 (5)	1 (4)	2 (5)	
Unknown	2 (3)	0	2 (5)	
HIV status				
HIV-positive	30 (49)	12 (48)	18 (50)	.95
HIV-negative, on PrEP	20 (33)	8 (32)	12 (33)	
HIV-negative, not on PrEP	11 (18)	5 (20)	6 (17)	
Among HIV-positive participants				
Plasma HIV RNA <20 copies/mL	26 (90)	11 (92)	15 (88)	1.00
Among HIV-positive participants, CD4 cell count/mm^3^	467.5 (335.75–677.75)	368 (327.5–819.25)	499 (382–659.25)	.58
Days since symptom onset at baseline	7.5 (5–13.25)	15 (9–17)	6 (3–7)	<.001
Days from symptom onset to resolution^c^	22 (17–34.5)	29 (19–36.5)	19 (16.5–27)	.1
Symptoms present at baseline				
Fever	38 (59)	17 (68)	21 (54)	.31
Headache	29 (45)	19 (76)	10 (26)	<.001
Lymphadenopathy	36 (56)	17 (68)	19 (49)	.20
Pharyngitis	22 (34)	11 (44)	11 (28)	.28
Proctitis	23 (36)	12 (48)	11 (28)	.12
Anogenital skin lesions	50 (78)	19 (76)	31 (80)	.76
Symptoms ever reported during illness				
Fever	43 (67)	17 (68)	26 (67)	1.00
Headache	34 (53)	19 (76)	15 (39)	.005
Lymphadenopathy	45 (70)	17 (68)	28 (72)	.78
Pharyngitis	24 (38)	11 (44)	13 (33)	.44
Proctitis	25 (39)	13 (52)	12 (31)	.12
Anogenital skin lesions	52 (81)	19 (76)	33 (85)	.51
Hospitalized during illness	4 (6)	2 (8)	2 (5)	.64
Smallpox vaccination before 2022				
None	39 (61)	16 (64)	23 (59)	.77
Yes	14 (22)	6 (24)	8 (21)	
Unknown	11 (17)	3 (12)	8 (21)	
MVA-BN vaccine before baseline^d^				
None	44 (79)	19 (79)	25 (78)	.19
1 dose	10 (18)	3 (13)	7 (22)	
2 doses	2 (4)	2 (8)	0	
Unknown				
Received tecovirimat during illness	20 (31)	9 (36)	11 (28)	.59

Abbreviations: IQR, interquartile range; MPOCS, Mpox Prospective Observational Cohort Study; MVA-BN, Modified Vaccinia Ankara-Bavarian Nordic; PrEP, pre-exposure prophylaxis.

^a^Values are median (IQR) or frequency (%).

^b^Comparing MPOCS and case series groups.

^c^Clinical resolution defined as the time when the scab falls off to reveal fully epithelialized skin underneath.

^d^Only 4 participants had received at least 1 dose of vaccine 14 or more days before symptom onset. Four participants who received 1 dose of vaccine (1 cohort participant, 3 case series participants) received the vaccine after symptom onset; all others were before symptom onset.

MPOCS participants provided a total of 666 samples over a median (range) of 4 (1–7) weekly visits, over a median (range) of 25 (1–72) days, with 22/25 (88%) completing the study 1 week after resolution of symptoms per protocol, and 3/25 (12%) lost to follow-up before that point; these 3 participants contributed 1, 3, and 3 visits, respectively. Case series participants contributed 204 specimens collected over a median (range) of 2 (1–4) visits, or 2 (1–26) days.

**Figure 1.**
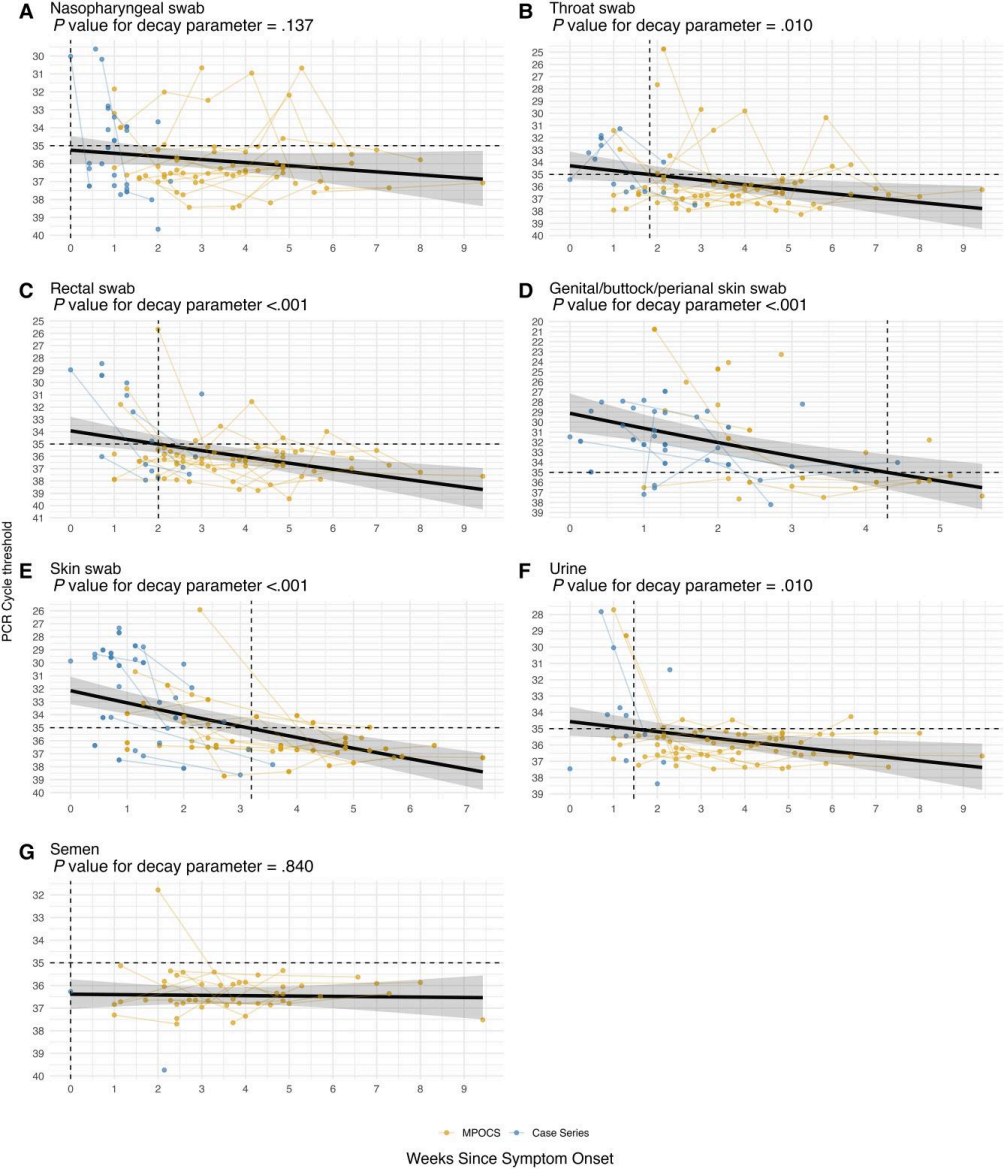
Mpox E9L computed tomography values by time since symptom onset among 64 men with mpox infection for 7 specimen types: (*A*) nasopharyngeal swabs, (*B*) throat swabs, (*C*) rectal swabs, (*D*) swabs from genital/buttock/perianal skin, (*E*) swabs from other skin sites, (*F*) urine, (*G*) semen. Vertical dashed lines indicate estimated time to resolution of detectable viral DNA. Abbreviation: MPOCS, Mpox Prospective Observational Cohort Study.

The estimated time in days from symptom onset to resolution of viral DNA detectability (Ct ≥ 35) varied by site and was highest for cutaneous lesions in the genital/perianal/buttock regions at 30 (95% CI, 23–47.9) days, and for other skin sites at 22.4 (95% CI, 16.6–29.4) days. Because a high proportion of participants had Ct values >35 at baseline from nasopharyngeal swabs and semen samples (Figure 1), the estimated time to resolution for these specimens was 0 (Table 2). Sensitivity analyses excluding participants contributing data on only a single date for a given specimen type showed similar results, but the median time to resolution was attenuated for nongenital/buttock/perianal skin, urine, and rectal swabs (Supplementary Table 1).

**Table 2.** Estimated Number of Days (95% CI) From Symptom Onset to Resolution of MPXV DNA Detectability (Ct Value ≥35)

Specimen Type	All Participants, Censored at First Use of Tecovirimat if Applicable	Comparison According to Tecovirimat Use		
		Participants Never Receiving Tecovirimat	Participants Receiving Tecovirimat	*P* Value^a^
Nasopharyngeal swab	0 (0–12.1)	0 (0–10.9)	0 (0–7.0)	.30
Pharyngeal swab	12.8 (0–24.9)	11.5 (0–24.9)	0 (0–19.8)	.22
Rectal swab	14.1 (0–22.4)	13.4 (0–21.7)	29.4 (0–N/A)	.50
Genital, buttock, or perianal skin	30.0 (23.0–47.9)	28.1 (23.0–37.1)	24.9 (19.2–35.8)	.18
Skin – all other sites	22.4 (16.6–29.4)	23.0 (17.3–29.4)	21.7 (0–40.3)	.24
Urine	10.2 (0–21.1)	10.2 (0–20.5)	0 (0–17.9)	.41
Semen	0 (0–0)	0 (0–0)	0 (0–15.3)	.73

Abbreviations: Ct, cycle threshold; MPXV, monkeypox virus.

^a^ *P* values correspond to the interaction between tecovirimat use and days since symptom onset in mixed log-linear models of Ct value as a function of time since symptom onset.

The proportion of participants with detectable viral DNA at baseline varied by sample type and was highest at 74% for genital/buttock/perianal skin and 56% for other skin specimens, down to a low of 8% for semen specimens (Table 3). The proportion of participants with detectable viral DNA declined over time for all sample types; among cohort participants who were followed until 1 week after complete clinical resolution and not exposed to tecovirimat, MPXV DNA persisted at Ct values <35 in 46% of genital/buttock/perianal skin swabs and 23% of other skin specimens (Table 3).

**Table 3.** Summary of MPXV DNA Detectability Findings by Sample Type

Specimen Type	No. (%) of Participants With Detectable MPXV DNA		
	At Baseline Visit (Among All Participants With Available Data)	At Final Visit (Among n = 22 Cohort Participants With Complete Follow-up Only)	At Final Visit (Among n = 14 Cohort Participants With Complete Follow-up Only and No Exposure to Tecovirimat)
Nasopharyngeal swab	12/46 (26)	4/22 (18)	1/14 (7)
Pharyngeal swab	13/35 (37)	4/22 (18)	2/14 (14)
Rectal swab	16/36 (44)	2/22 (9)	0/14 (0)
Genital, buttock, or perianal skin	31/42 (74)	6/18 (33)	5/11 (46)
Skin – all other sites	27/48 (56)	5/19 (26)	3/13 (23)
Urine	10/37 (27)	1/22 (5)	1/14 (7)
Semen	2/25 (8)	0/21 (0)	0/14 (0)

Abbreviation: MPXV, monkeypox virus.

We next examined the relationship between mucosal symptoms and viral DNA detectability. In participants reporting pharyngitis during their illness, the proportions with detectable viral DNA in pharyngeal swabs and nasopharyngeal swabs at baseline were 4/12 (33%) and 7/19 (37%), respectively. These proportions were not significantly different from the corresponding values for participants not experiencing pharyngitis symptoms, who accounted for 9/23 (39%; *P* = 1) and 5/27 (19%; *P* = .19), respectively. However, the proportion of participants with detectable viral DNA in rectal swabs at baseline was significantly higher among participants ever vs never reporting proctitis symptoms, at 13/19 (68%) vs 3/17 (18%), respectively (*P* = .003).

**Figure 2.**
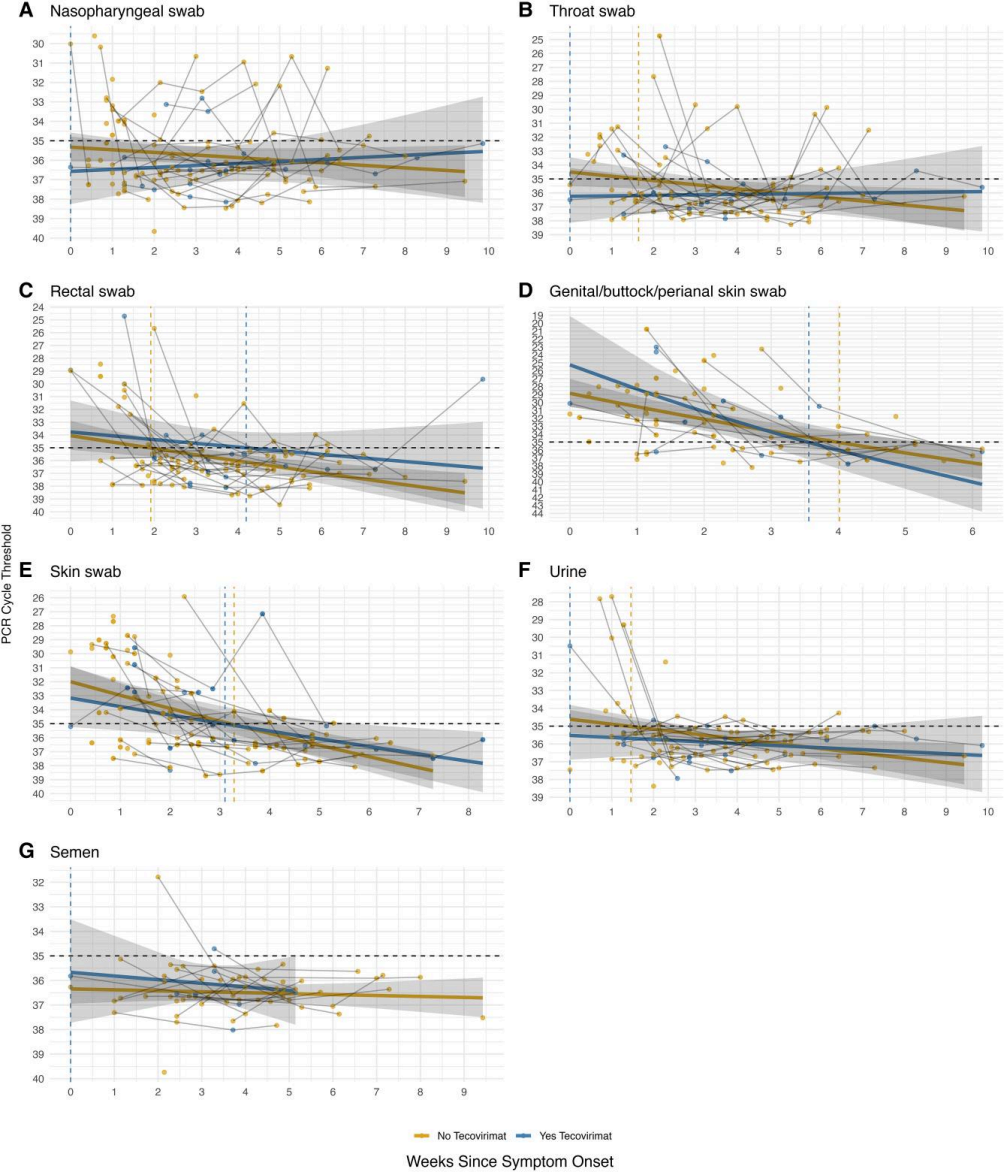
Mpox E9L computed tomography values by time since symptom onset according to receipt of tecovirimat for 7 specimen types: (*A*) nasopharyngeal swabs, (*B*) throat swabs, (*C*) rectal swabs, (*D*) swabs from genital/buttock/perianal skin, (*E*) swabs from other skin sites, (*F*) urine, (*G*) semen. Abbreviation: PCR, polymerase chain reaction.

Tecovirimat was administered to 20/64 (31%) participants due to symptom severity and/or anatomic site of involvement (including 1 case each of myocarditis, esophagitis, and conjunctivitis). Treatment was initiated a median (IQR) of 8 (12–21) days after symptom onset in these 20 individuals. For all specimen types examined, our LMER models of Ct value over time showed no statistically significant evidence of an interaction between tecovirimat use and days since symptom onset, suggesting that there was no important difference in the rate of decay in Ct value among those receiving vs not receiving tecovirimat (Table 2, Figure 2).

## DISCUSSION

In this prospective observational sample of 64 men with mpox, viral DNA persisted at Ct levels <35 for up to 30 days after symptom onset. The duration of viral DNA detection varied by specimen type, with the longest period seen in samples from genital/perianal/buttock skin, at just over 4 weeks. Viral DNA detectability lasted roughly 3 weeks in other skin specimens, 2 weeks in throat and rectal swabs, 10 days in urine, and was negligible in nasopharyngeal swabs and semen. These findings may be useful for interpreting MPXV PCR tests over the course of illness and reinforce the utility of collecting diagnostic specimens even when patients present to clinical attention days or weeks after symptom onset. Our results may also inform the understanding of mpox transmission.

Our results are broadly consistent with other studies. In a German case series of mpox patients with acute illness, MPXV DNA was highest, and remained detectable for longest, in skin lesions compared with oropharyngeal swabs and blood, although only 5 individuals were included and formal statistical testing was not performed [15]. Another case series demonstrated that DNA MPXV loads could be detected not only from skin lesions and nasopharynx swabs over time but also from exhaled breath using facemasks, as late as 31 days after symptom onset, although only 2 participants were included [26]. The 2 largest published longitudinal studies included sample sizes similar to ours. Among 50 men in Paris, the proportion of samples with detectable MPXV DNA varied by specimen type, at 88% (skin), 71% (anal swabs), 77% (throat), 22% (urine), and 54% (semen) at baseline, and fell to 22%, 9%, 0%, 0%, and 9%, respectively, at day 14 [16]. Among 77 adults in Spain, the median time to clearance of MPXV DNA by PCR was very similar to what we observed and was highest in skin (25 days) compared with the oropharynx (16 days) and rectum (16 days) [17]. A novel finding from our analysis was the longer duration observed for genital/buttock/perianal skin (30 days) compared with other skin sites (22 days), which we speculate to be the result of a higher burden of virus at sexually exposed anatomic locations; this finding may not be expected to hold for individuals who acquire mpox through other modes of transmission.

Notably, the Spanish study described above observed a longer duration of viral DNA in semen than we did, at 13 days [17]. Possible explanations include differences in laboratory techniques, specimen collection methods, or the number of participants providing semen samples (65 vs 21 in our study). Further, MPXV DNA levels may not change in a monotonic fashion in semen and/or in other specimen types, which was a simplifying assumption in our models. In a systematic review on MPXV DNA detectability in semen samples, semen PCR testing was positive in 84/643 (13.1%) of confirmed mpox cases, although the number of people tested, the participants’ stage of illness, and the Ct threshold used to define PCR positivity were not reported [27]. In a separate review regarding the PCR positivity rate in semen, a meta-analysis of 5 studies yielded a semen positivity rate of 72.4% (95% CI, 55.7%–84.5%), although again the timing of specimen collection and the Ct value cutoff were not specified [28]. Among 3 patients whose PCR-positive semen samples were tested, only 1 showed cytopathic effects consistent with viral replication competence [19, 28, 29].

In our study, viral DNA was more often detected in rectal swabs among those experiencing proctitis, similar to a small American study that detected MPXV DNA in 4/4 mpox patients with proctitis but in 0/3 of those without proctitis [30], but a comparable relationship was not observed between throat or nasopharyngeal swabs with pharyngitis symptoms. Several participants had detectable virus in these specimens in the absence of localizing symptoms, consistent with prior reports of asymptomatic MPXV DNA detectability in oropharyngeal and anorectal swabs [31, 32], and with other studies indicating low viral DNA positivity in oral and anal samples [22].

The relationship between detectability of MPXV DNA, viral replication competence, and transmissibility remains unclear, although several studies have attempted to identify PCR Ct thresholds that correlate with viral viability. Among 174 samples from Spain, evidence of viral replication was seen in 70% of 47 samples with viral loads ≥6.5 log10 copies/mL (roughly corresponding with Ct values ≤26) and none of those with lower viral loads [17]. Among 41 saliva samples and 45 medical masks worn for 30–45 minutes from 44 patients in Madrid, viable virus could only be detected in samples with Ct values below about 27 and 30, respectively [23]. In a longitudinal study of 54 samples from 4 patients in the United Kingdom before the 2022 outbreak, viral cytopathic effect was only observed in samples with Ct values ≤31.0 [24]. Among 43 specimens from 32 patients in Israel, the estimated Ct threshold corresponding to the lower limit of detection for viral infectivity was 34.98 [21]. An Australian study reported that the Ct value at which 50% of PCR-positive clinical samples were positive in viral culture was 34.1 (95% CI, 32.1–37.4) [22]. Our selected Ct threshold of ≥35 for defining clearance of viral DNA was based on these findings.

We observed no significant difference in the rate of decay in detectable MPXV DNA in participants receiving tecovirimat compared with others. These findings are consistent with a recent report on 41 patients with mpox, of whom 19 received tecovirimat, in which an emulated target trial suggested no difference in healing or time to viral clearance [33]. However, in both studies, tecovirimat use was based on clinician judgment and drug availability rather than randomization, treatment was initiated over 1 week after illness onset, and sample sizes were small. Randomized trials comparing tecovirimat and placebo for mpox are ongoing [34, 35], and enrollment in these studies should be prioritized where possible. In a case series of 3 individuals with severe mpox treated with tecovirimat, viremia was high compared with a larger cohort of patients with less severe disease and declined by a median of 2.21 log over 1 week, but viral DNA load was more variable in other compartments [36].

The strengths of our study include the longitudinal specimen sampling and the breadth of specimen types evaluated and the inclusion of patients representing a range of disease severity. Our study also has limitations. First, we combined data from 2 studies, using 2 different MPXV DNA PCR assays. To mitigate the impact of this difference on our models, we transformed PCR data from the case series participants using linear regression to facilitate comparability with results from the cohort participants. Second, our analyses focus on Ct values, which are not standardized units of viral burden. Third, our modest sample size precluded our ability to draw robust conclusions on the importance of clinical characteristics such as mucosal symptoms and tecovirimat use. Finally, we lack data on viral replication competence, and our PCR Ct threshold for defining MPXV DNA detectability has been associated with reduced, but not 0, infectivity in prior studies.

In conclusion, we found that adults with mpox continue to have detectable viral DNA for an average of up to 30 days after symptom onset, with the duration varying by specimen type. Although we defined DNA detectability using a Ct threshold that roughly corresponded to viral infectivity in prior studies, correlation with mpox transmissibility requires further study.

## Supplementary Material

ofae073_Supplementary_Data

## Supplementary Data

Supplementary materials are available at *Open Forum Infectious Diseases* online. Consisting of data provided by the authors to benefit the reader, the posted materials are not copyedited and are the sole responsibility of the authors, so questions or comments should be addressed to the corresponding author.
