# Supplementary material for: Longitudinal Analysis of Mpox Virus DNA Detectability From Multiple Specimen Types During Acute Illness: A Cohort Study
Source: Open Forum Infect Dis. 2024 Feb 7;11(2):ofae073. doi: 10.1093/ofid/ofae073 (PMC10883290; doi:10.1093/ofid/ofae073)
Supplement: ofae073_Supplementary_Data [file ofae073_supplementary_data.docx]

**Longitudinal analysis of mpox virus DNA detectability from multiple specimen types during acute illness: a cohort study**

**Supplementary Materials**

**Figure S1. Participant flow diagram**

**
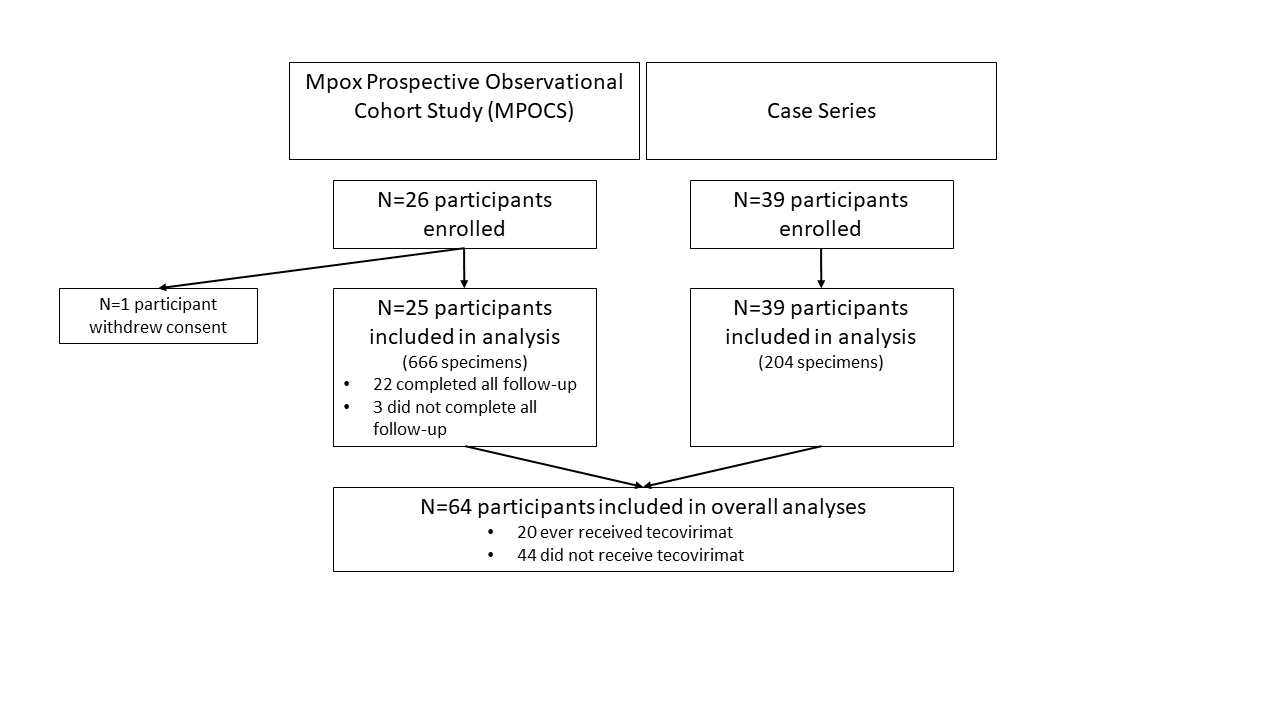
**

**Table S1. Sensitivity analysis showing estimated number of days (95%CI) from symptom onset to resolution of MPXV DNA detectability (Ct value ≥35), restricted to participants with two or more visits and censored at first use of tecovirimat if applicable**

| **Specimen type** | **Primary analysis** | | **Sensitivity analysis** | |
| --- | --- | --- | --- | --- |
|  | **Days** | **n** | **Days** | **n** |
| Nasopharyngeal swab | 0 (0, 12.1) | 42 | 0 (0, 8.3) | 21 |
| Pharyngeal swab | 12.8 (0, 24.9) | 31 | 7.0 (0, 25.6) | 21 |
| Rectal swab | 14.1 (0, 22.4) | 32 | 0 (0, 12.8) | 21 |
| Genital, buttock, or perianal skin | 30.0 (23.0, 47.9) | 38 | 30.0 (23.6, 41.5) | 14 |
| Skin – all other sites | 22.4 (16.6, 29.4) | 42 | 12.1 (0, 19.2) | 18 |
| Urine | 10.2 (0, 21.1) | 32 | 1.3 (0, 17.3) | 21 |
| Semen | 0 (0, 0) | 21 | 0 (0, 0) | 19 |
